# Acoustic Biotopes, Listeners and Sound-Induced Action: A Case Study of Operating Rooms

**DOI:** 10.3390/ijerph192416674

**Published:** 2022-12-12

**Authors:** Elif Özcan, Cornelis L. H. Broekmeulen, Zoe Alexandra Luck, Monique van Velzen, Pieter Jan Stappers, Judy Reed Edworthy

**Affiliations:** 1 Critical Alarms Lab, Faculty of Industrial Design Engineering, Delft University of Technology, 2628 CE Delft, The Netherlands; 2 Department of Anaesthesiology, Leiden University Medical Center, 2333 ZA Leiden, The Netherlands; 3 Department of Human-Centered Design, Faculty of Industrial Design Engineering, Delft University of Technology, 2628 CE Delft, The Netherlands; 4 School of Psychology, University of Plymouth, Plymouth PL4 8AA, UK

**Keywords:** acoustic biotopes, sound environments, sound-action coupling, listening types, operating rooms

## Abstract

As socio-technological environments shape and direct listener behaviour, an ecological account is needed that encompasses listening in complexity (i.e., multiple listeners, multiple sounds and their sources, and multiple sound-induced actions that ensure the success of a mission). In this study, we explored sound-induced action under the framework of “acoustic biotopes” (a notion of ecological acoustics by Smolders, Aertsen, and Johanessma, 1979 and 1982) in a specific socio-technological environment, i.e., the context of an orthopaedic operating room. Our approach is based on literature research into the topics of environmental psychology and auditory perception and action and in situ observations in healthcare with field recordings, participatory observations, and interviews on the spot. The results suggest a human-centered definition of sound-induced action in acoustic biotopes: Acoustic biotope is an active and shared sound environment with entangled interactions and sound-induced actions taking place in a specific space that has a critical function. Listening in highly functional environments is an individual experience and is influenced by hearing function, physical position and role in an environment, and the task at hand. There is a range of active and passive sound listeners as a function of their attentive state and listeners as sound sources within the acoustic biotope. There are many different sound sources and sound locals in socio-technological environments and sounds have great potential to serve critical information to operators. Overall, our study provides a holistic, multi-layered and yet a listener-centric view on the organisation of complex spaces and the results can immediately be applicable for rethinking the acoustic environment for ORs for better listening and sound-induced action.

## 1. Introduction

In this paper, we explore the notion of a listener-centric sonic environment and demonstrate with a case study how sound-induced action is dependent on who listens and what their role is in the context of highly functional ecosystems. Socio-technological environments such as mission control rooms, airplane cockpits or operating rooms cater for critical tasks and actions, and their sound environment is conducive to social, environmental, and instrumental interactions. For example, an alarm in a cockpit requires a pilot’s action to run a designated procedure while the co-pilot is on stand-by; or a nurse and a surgeon may communicate without words only on the bases of the mallet or incision sounds produced by surgical tools. Listening in such complex environments is central for taking the required actions in a timely manner and listeners become actors with a task to accomplish. In professional ecosystems, collaborative work relies on individuals’ own appraisal of sound events [1,2] as different listeners would have different concerns deriving from their own tasks at hand [3]. Collective listening and listening in networks have also been explored by Paine, Bevilacqua, and Matuszewski [4] in a recent Special Issue on Collective and Networked Sound practices. As socio-technological environments shape and direct listener behaviour, an ecological account is needed that encompasses listening in complexity (i.e., multiple listeners, multiple sounds and their sources, and multiple sound-induced actions that ensure the success of a mission).

Our exploration will expand the earlier work on sound-centric studies (e.g., psychoacoustics, product sound quality, soundscape perception) that focus on sound being the cause of more generic human experiences (i.e., from perception and cognition to action) and imply that sound or its source can and should be manipulated for improved perceptual and cognitive experiences [5,6,7,8]. This approach has indeed been so far instrumental in engineering and design of products and (eco)systems for improved functionality and acceptability. However, in recent studies, delle Monache [9,10] showed that listening-in-action is the primary and core activity when either using sound for completing a task or designing for human-sound interactions. User-centered approaches emphasize the importance of understanding the context of the user and their needs for better designs (e.g., Sleeswijk Visser and Stappers’s work on context mapping [11]). Similarly, our listener-centric approach for complex functional environments is intended to highlight listeners and advocate that the listening experience is in the ‘ear of the beholder’ and is defined by listener’s goals with the product/service/system.

The phenomenon that complex sound environments offer different possibilities for action has been studied about 40 years ago in the field of ecological biology and perception focusing on the ecosystem of animal-sound-environment trilogy [12,13]. The term “acoustic biotope” was coined to describe how a particular sound (i.e., acoustics) in a habitat (i.e., biotope) represents different meanings and action possibilities for different species for their survival. The study of sound phenomena in diverse ecologies (e.g., Schafer’s seminal work [14]) has recently become more popular addressing notions of soundscapes, acoustic ecology, ecoacoustics, bioacoustics, and acoustic diversity [15,16,17] in relation to how different species utilise sound for daily functions and inter- and intra-species communication in large habitats. In order to support successful workflows and collaboration in professional environments, we draw similarities between species behaviour in habitats and listener behaviour in socio-technological environments and embark to understand the role of listening in the dynamics of human-system and human-human interactions.

### This Paper and the Use Case

In this paper, our aim is to position acoustic biotopes as a novel concept to focus on listener behaviour in socio-technological sound environments and explain it with the relevant touchpoints which are to be defined in this study. A case study in orthopaedic operating rooms (ORs) is presented to be able to draw conclusions from real life examples as surgical procedures in ORs require the most sound-producing medical equipment in healthcare contexts. For example, the sound of a mallet allows positioning the prosthetics, scrub nurses prepare surgical tools for when the drilling stops, music motivates a surgeon who hits the mallet to the music’s beat, a repetitive beep alerts an anaesthesiologist, lack of sound signifies suction-pump malfunctioning.

Our approach is based on literature research into the topics of environmental psychology and auditory perception and action and in situ observations with field recordings, participatory observations, and interviews on the spot. While we primarily aim to expand the knowledge domain of sound perception and action in complexity, the findings will also benefit the healthcare domain by creating awareness regarding the potential of sound for interactions in surgeries and introduce the possible roles of OR listeners in the surgical workflow. The OR sound environment is typical of socio-technological environments: unorganised, cacophonous, potentially harmful for wellbeing, and can even damage hearing. There is an active call to improve the sound environments of critical care [18]. Thus, understanding how listening functions in complexity will also urge the field of design engineering and the management of highly functional workspaces to respond to the listening needs of expert users eventually organizing sound events and procedures around listening.

## 2. Background into Listening in Complex Sound Environments

### 2.1. OR as a Rich Sound Environment

**Figure 1:**
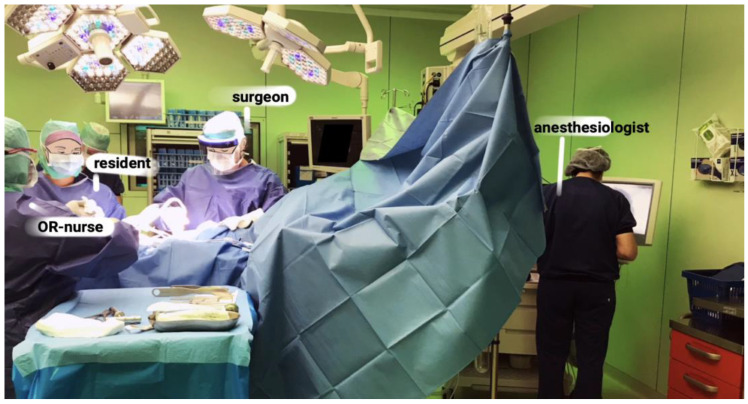
The setting of an orthopaedic surgery.

The Operating Room (OR) is a highly specialized medical working environment, in which different actors work together on the surgery of a patient with the support of specialized tools and devices (See Figure 1). These tools, devices and actors create a range of sounds, e.g., music at the background, mallet or drilling sound around the patient, alarms beeping in the corner and OR-staff talking and moving around next to the constant noise of suction-pumps and mechanical-ventilators. It is widely acknowledged that OR sounds create excessive noise and are hitherto considered as harmful to clinicians’ wellbeing and health [19,20,21,22,23,24,25].

In the orthopaedic OR, several instruments (mallets, oscillating saws, drills) produce sound within the range of 95–106 dBA (sound levels similar to a train passing at 70 km/h) and with peak levels of 120–140 dB experienced more than 40% of the time [26,27]. OR-staff behaviour produces sounds too [27], e.g., preparing for operation, moving trolleys/equipment, opening/slamming doors, using/dropping metal tools, performing suction or talking loudly. OR-staff are also sound receivers. The sound of dropping a tool will most likely alert at least one staff member and initiate action, for instance picking up and exchanging the specific tool with a sterile one. Non-behaviour-related environmental factors (e.g., anaesthetic curtain) influence sound interactions [28] and when visual communication/interaction is restricted, the need for auditory information grows correspondingly [29].

It is well established that excessive noise is a problem in clinical care and the orthopaedic OR is a prime example of an environment that is non-optimal in terms of noise levels [30,31,32,33,34,35,36,37,38,39,40,41,42]. This will affect the wellbeing, ability to communicate, and other aspects of the interactions between surgical staff, which are already integrated into the communication and interaction patterns with the surgical OR as this paper demonstrates. The more general problem of excessive noise in the OR is not a key focus of this paper per se, although it is alluded to on several occasions. Excessive noise in the OR is known to be a problem which has no simple solution, but there are many steps which can be taken to reduce the noise such as the redesign of instruments, the reduction of false alarm rates and the redesign of auditory alarms, and the room layout and materials used to make surgical tools and objects (see [43,44,45,46] for good practices to overcome unwanted sounds in healthcare). The key focus of this paper is however how sound is used, how the individuals interact with the sound differently, and how the sounds have different roles for the different individuals. Listening and action take place within a cacophonous environment which needs to be studied.

### 2.2. Listeners and Sound Environments

Humans have a great ability to capture rich information from sound events (e.g., distinguishing pouring hot/cold water in a cup, knowing when the cup is full). Specialist listeners are further trained to use sound to the benefit of their work (e.g., astronauts, motorists). Since the 1960s, our understanding of how humans listen has evolved and researchers found ways of categorising them [2,47,48,49]. We summarise these notions from Schaeffer, Chion and Gaver into three main categories based on their similarities: Sound-oriented listening (focusing on the acoustic phenomenon and components), source-oriented listening (discovering the object as sound source), and meaning-oriented listening (how the identified sound represents certain socio-cultural and emotive concepts). These accounts also reflect the intentionality of listeners and their relevance to their current situation. Listeners may intend to construct the causal relationship between the sound and its source, to understand the environment and its significance through sounds, or they may want to assess the acoustic qualities of the sound itself. When sounds become functional entities in an environment, listening attention plays a role in completing tasks and ranges from focused listening (i.e., listening-in-search) to a more general scan of the environment (i.e., listening-in-readiness) and just passively hearing the environment (i.e., background-listening) [50]. Tuuri & Eerola’s more recent study [2] offers a more holistic account to listening and describes how listening is embedded in the situation and context also pointing out that listening function guides, and is guided by, socio-physical contexts. Thus, what people hear depends on who listens and how people listen influences possibilities for interaction with the different components of an environment.

A majority of daily sound experiences are implicit and yet there is a direct relation between sound perception and action. Listeners can estimate the size, shape, weight, location, and movement of an object through sound [51,52,53,54,55,56,57,58,59,60,61,62,63] and their actions are accompanied by informative sounds through continuous acoustic feedback (e.g., estimating the size/distance of a car approaching) [64,65]. Tuuri and Eerola [2] emphasised the role of listening in the trajectory from perception to action and made a distinction between three sound-action couplings (AKA, sound-induced action as referred in our current study): We interpret their ‘action-sound-object’ as sound-induced action learnt by experience; ‘action-sound-intersubjectivity’ as sound-induced action interpreted differently by listeners; and ‘action-sound habit’ as predictable sound-induced actions in familiar situations. Research also highlighted the embodied responses towards heard sounds and thus exemplified reflexive actions such as altering bodily movement (e.g., altering walking sounds improves one’s gait; music guides (elderly) physical exercises) [66,67,68].

**Figure 2:**
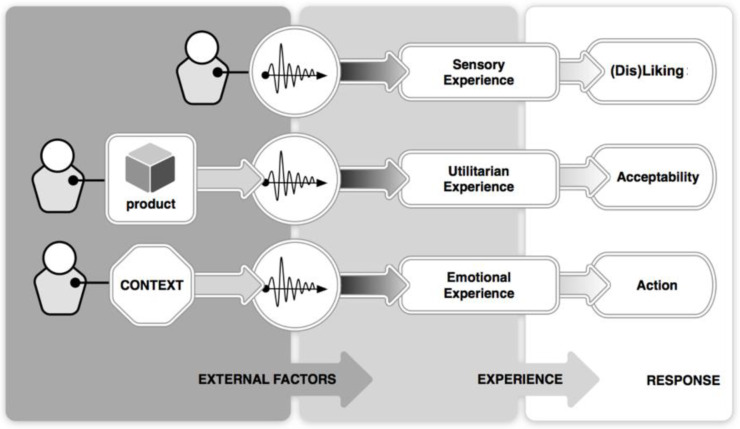
Product sounds evoke different experiences (sensory/utilitarian/emotional) depending on external factors (Adapted from [69]).

Recent studies of delle Monache and colleagues [9,10] also demonstrated the permanence of sound-action coupling in the way people express their experiences with sounds. In previous research, Özcan [69] demonstrated the varying degrees of sound experiences ranging from sensory (dis)pleasure to functional experience of products through sound and to how context shapes users’ emotional responses towards product sounds (see Figure 2) [70,71,72,73]. The conclusions of this research were complementary to our notion of environmental sound perception (e.g., alarms, machinery sounds, music, speech) [74,75,76,77,78,79]. Sounds in an environment can be pervasive and experienced also collectively [80,81,82,83,84]. Schokkin et al. [85] found that intensive care nurses expressed their individuality through sound and these expressions collectively fitted in three sound-conscious subcultures and fitting behavioural patterns (assertive ally, opinionated professional, docile novice).

Sound events in real life are rarely experienced in isolation and are always part of an eco-system that contains objects and people that produce the sound. Thus, the experience of sound environments, (also known as, soundscapes) entails psychological analysis of a collection of sounds (e.g., sounds of a rainforest with wind, leaves, birds and animals, urban soundscapes with vehicles passing, people talking, and weather sounds) [14,80,81,82,83,84,86] with listeners’ physiological characteristics considered such as hearing capacity of the receiver and the orientation or position of listening considered [15]. Axelsson, Nilsson, and Berglund [87] offered a three-dimensional space based on psychological evaluations and physical (i.e., acoustical) analysis of a range of Swedish soundscapes. Hence, soundscape experience was reduced to Pleasantness, Eventfulness, and Familiarity/Appropriateness dimensions. For indoor soundscapes, Torresin and colleagues [88] showed that outdoor sounds still dominated the indoor soundscape experiences which are described as Comfort, Content, and Familiarity similar to Axelsson et al.’s dimensions. However, more specific to indoor soundscapes, Engagement and Privacy—Control were found to be two relevant descriptive dimensions representing listeners’ wish to have a sense of control and engagement with sound events in more private settings. These results also suggest that listeners of indoor soundscapes, such as ORs, will require more possibilities for sound-induced action as there seems to be a tendency to engage in indoor sound events and control their occurrence as well. The psychological dimensions of sound environments were further extended in a review article by Aletta, Kang, and Axelsson [89] by extending the focus on the effects on the listener and adding components such as Restorativeness, Tranquillity, and Annoyance. Remaining dimensions still refer to a more technical and psychoacoustical assessment of soundscapes such as Sensory Pleasantness, Music-likeness and Quality Assessment. The effect of context is also considered in Aletta et al.’s study as appropriateness of the sound events to the location/situation in question. It is also important to acknowledge that architectural setups and different tasks at hand may differently influence the way listeners perceive the acoustic environment, manage expectations by prioritising needs, and take consequent actions. For our study, context is a major component to focus on, because possibilities for sound-induced action can only be increased if listeners are able to make sense of the sound events in a particular environment that has ecologically a specific function. Below, we will extend this notion further.

### 2.3. From Acoustic Space to Bio Acoustic Space and Acoustic Biotopes

**Figure 3:**
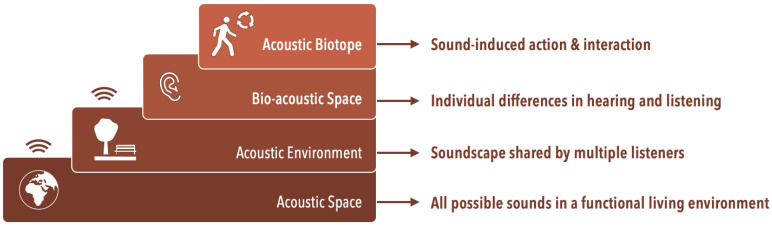
The relationship between the entire acoustic space which encompasses all possible sounds and acoustic biotope which encompasses sounds that can be captured by species in a functional living environment and are directly related to the species.

In the field of biological/ecological neuroscience, Johannesma and Aertsen [12] categorize the patterns of sound in an environment in relation to the meanings associated with action possibilities for certain species that inhabit that environment. While they have a computational account of probabilities and possibilities of occurrence of sound within the realm of the species, their categorization offers us possibilities to have a converging perspective into the varying degrees of interactions with environmental sounds. Below we will elaborate on their definitions: acoustic space, acoustic environment, bioacoustics space, and acoustic biotope. Figure 3 provides a representation of the hierarchical relation between these different environmental sounds and the species in question. The figure also connects to the conceptual framework described in the ISO 12913-1 standard [90], in which the constructs of ‘acoustic environment’, ‘soundscape’ and ‘context’ are conceptually intertwined.

**Acoustic Space** contains all possible sounds that are present in the biotope.

**Acoustic Environment** provides an account of the auditory patterns that may carry information relevant to a member of a species (e.g., meanings in the sense of predators to avoid, prey to catch, etc.). Hence, arbitrariness or permanence of sounds can be established within an acoustic environment. Rainforests have a certain acoustic environment that will most likely not include automobile sounds and urban acoustic environment will unlikely offer choruses of crickets, but when sounds naturally occur in the habitat they belong to, they become meaningful and instrumental to the species of that habitat.

**Bio-Acoustic Space** contains all sounds that can possibly be captured by a certain species. For example, the acoustic environment of a duck and frog may be identical but not all sounds will evoke auditory imagery for a duck or for a frog. Auditory imagery indicates the relevance of the sound to the species as the meaning of the sound resides in the sound evoked imagery. Therefore, bio-acoustic space suggests the possibility that a sound is perceptually relevant to the listener’s hearing system. Furthermore, sensory- and neuro-physiology allows us to determine sensory capacity of the species and their ability to perceive a sound. Thus, it is important to understand and study the perceptual capacity of the listener.

**Acoustic Biotope** considers the general aspects of the behaviour of the species (e.g., position, locomotion, interaction possibilities) and how their sound-induced behaviour influences their actions within a specific environment (Figure 3). A biotope is a local environment in which a set of species resides as actors, and which carries information that guides these actors through structure of (light and) sound. Animals have evolved a sensitivity to sounds of approaching predators or prey, environmental conditions like wind and rain, and vocalisations to inform each other (sometimes across species boundaries) of possible threats and opportunities. While biotopes are inherently a place for interaction within species, acoustic biotopes introduce a listener-centric inquiry within the environment of the species and offers the notion of sound-induced behaviour and actions amongst the members of the species [13].

### 2.4. Acoustic Biotopes of Operating Rooms

To explain the possible roles of sound and listeners in the OR, we build on the notion of acoustic biotope as used in ecological biology and perception. In our case, the orthopaedic OR is the biotope, the species are healthcare professionals that serve in the operating theatre, and the structured sound comes from instruments, the various bodies and actions of the actors (i.e., OR staff). Some of these sounds are explicitly designed to convey information (e.g., the beep of a heart monitor), others are part of normal functioning (sounds of actions, of breathing, of posture changes) and may be picked up in subtle ways, much in the way an automobile driver uses the sound of the car’s engine, or from neighbouring vehicles. Thus, the OR sound environment allows for the acoustic biotope in which multiple listeners, multiple sounds, a variety of objects, are all bound by the sound environment and interactions with each other happen upon capturing subtle information from sounds.

**Figure 4:**
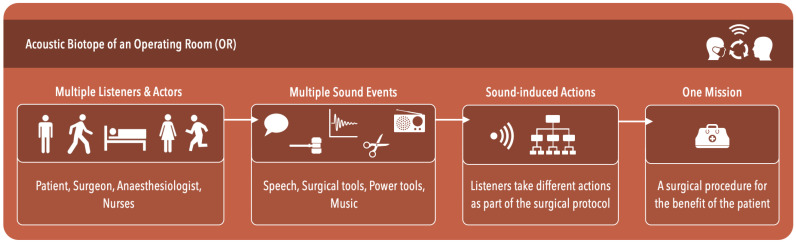
Basic representation of the acoustic biotope of an OR. The ultimate goal of an acoustic biotope is to facilitate sound-induced actions and interactions amongst the OR team members.

In this notion, although listeners (i.e., surgeons, nurses) are considered to possess biologically identical hearing systems (i.e., human ear), these actors within the biotope will have individual differences in the way they pick up sound, listen, process sounds and find a sound’s relevance to their own action [47,48,49]. Similar to different species such as frogs and ducks, we can work from the premise that different parts of the auditory signals may carry different meanings for different actors, depending both on their hearing ability, but also on their repertoire of possible actions (and hence, the information that may guide them in these actions). Such individual differences may depend on gender, age, experience, role and physical position in the OR. Previous research of Deb and Claudio [91,92] has also highlighted the individual differences amongst critical care nurses in the way they interpret medical alarms. In Figure 4, we organize the acoustic biotope of an OR converging from hospital sounds to sound-induced action which the OR team needs to individually take during the surgery.

## 3. Observational Study in an Orthopaedic OR

### 3.1. Method

In this section, we set out to understand the main elements for the acoustic biotope of an operating room based on in situ observations and recordings. We have observed 13 (6 + 7 at different times) orthopaedic surgeries (total hip replacement) each lasting in average 30 min in a private clinic in the Netherlands. We collected sound intensity levels in dB and audio/video recordings (six out of 13 observations resulted in dB(A) (LAeq) measurements and seven out of 13 resulted in dB(C) (LCpk) measurements). For the dB measurements, we used a calibrated Bedrock SM30 class 2 measuring instrument with included BAMT2 microphone (20–20,000 Hz frequency range and dynamic range 24–124 dB), approximately 1.0 m away from the operative field. The sound levels measured in LAeq and LCpk are used as a rough indicators of perceived loudness level that would ideally be measured in phons or sones. In the remaining seven observations, we observed the dynamics of the OR team members, identified sound sources and had semi-structured interviews with OR team members to increase our knowledge of the OR setting. We used sound measurement tools and video recordings without violating the privacy of patients and OR team. All participants gave written consent to the observations. A permission has been obtained for an ethical conduct of our research on 14 April 2020 by TU Delft’s Human Research Ethics Committee.

**Figure 5:**
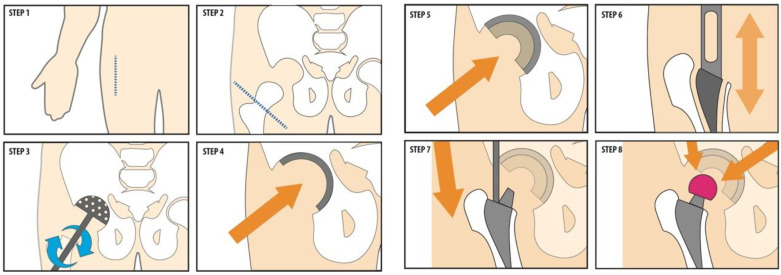
The procedural steps of Total Hip Arthroplasty (THA) Surgery. Step 1. Incision; Step 2. Cutting the Femoral neck; Step 3. Reaming the Acetabular; Step 4. Placement of Acetabular cup; Step 5. Placement of Acetabular cup liner; Step 6. Opening the Femoral canal; Step 7. Placement of Femoral stem; Step 8. Placement of Femoral head. The arrows indicate the force applied by the surgeon with a surgical hammer which as a consequence produces sound.

We observed a Total Hip Arthroplasty (THA) surgery. In total hip arthroplasty surgeries, the head of the femur and the socket part of the pelvis (acetabulum) of the natural hip get replaced. Hip procedures include first-time or primary joint replacement as well as revision of older implants. Within this project there is specifically research done on anterior approach procedures where the hip implants are press-fitted into the bone, which means that they get fixated to the bone through on-growth or ingrowth technology. The procedure is exemplified in Figure 5.

Our study will result in the acoustical description of the sound environment in terms of sound levels, a map of sound events and their location in the OR, task analysis of OR teams and the information flow amongst them, the potential of OR sounds to be used as surgical cue, subjective account of how OR sounds are experienced by the OR team members and how OR team members listen to their environment and switch attention and role whole listening. Our major contribution is to bring together all the psychophysical elements of OR sounds that guide listening within the notion of acoustic biotopes of operating rooms in order to facilitate teamwork and interactions.

### 3.2. Sound Measurements and Sound Sources

**Figure 6:**
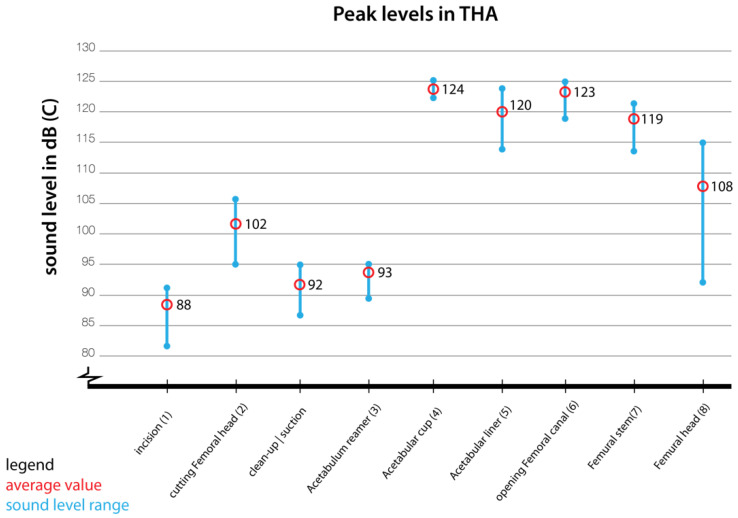
Peak dB(C)—LCpk measurements for (powered) surgical tools. Refer to Figure 5 to see the actions causing the sound.

During surgery an average sound level was measured in dB(A) (LAeq) and presented in Table 1. LAeq corresponds with sound levels that can be perceived by the human ear (time-average sound level). The average sound level measured during a surgery was 80 dB, this gives a total weighted average of 74 dB(A). We also individually observed the surgical tool usage as seen in Figure 5 and collected dB(A) data for each key surgical action (e.g., incision, oscillating saw, clean up/suction, rasping femur, etc.) in Figure 6. The quietest action was incision (range 57–67 dB(A)) and loudest action was rasping femur (85–102 dB(A)). We also observed that when music was played in the ORs, the dB-meter indicated 87 dB(A). We did not make special recordings of alarms sounds neither conversation. However, literature indicates that the intensity of medical alarms is registered at approximately 77 dB and the intensity of OR team conversations range from 61–68 dB [93].

We also measured the peak sound levels (LCpk) which correspond to C-weighted frequency sensitivity to allow for correct measurement of true peak sound levels (See Table 2). LCpk measurements helped us to understand the peak sound levels values of OR (powered) tools as they may potentially be harmful to the ear and cause pain. If pain is caused by sound, then tasks will also be influenced by that. Figure 6 shows the peak levels for each surgical task and also indicates how salient (i.e., noticeable) they might be. The sound level peaks within the total hip replacement procedure ranged from 116 to 127 dB(C). Average peak levels range between 88 dB(C) for incisions and 124 dB(C) for placement of acetabular cup. As hearing loss due to noise can be temporary, a reduced sensitivity to sound over a wider frequency range resulting from exposure to brief extremely loud noises (120 dB(A) or over) may cause permanent hearing loss [94]. As levels above this value do exceed the pain threshold of the human ear which is set around 120 dB [95]. Regular exposure to these high levels will results in noise induced hearing loss and tinnitus in the long-term. Placement of the acetabular cup and opening of the femoral canal account for the highest peaks and are around and above the human pain threshold of 120 dB. Therefore, we can conclude that surgical steps that include mallet and chisel blows are the most deafening because of the duration and the sound level reached (femur opening (123 dB(C) and acetabular cup (124 dB(C)). Masking also occurs due to medical alarms and background noise and causes serious problems for speech intelligibility and compromises interactions amongst the clinicians [96,97,98].

**Table 1:** Registered dB(A)—LAeq during surgeries.

dB(A)-LAeq	21.10/1	21.10/3	21.10/4	01.11/1	01.11/2	14.11/1	14.11/2	Range	Average (log)	Set Average
Incision	66.8	65.3	61.1	64.7	67.2	57.3	60.2	57–67	64.1	64
Saw (osc)	68.4	84.2	74.6	84.7	83.2	79.6	76.4	68-85	81.3	81
Clean-up/suction	72.2	65.8	64.6	66.6	63.2	62.6	69	63–73	67.7	68
Reamer (elec)	72.2	70.5	66.1	64.3	67.1	68.2	67.1	64–72	68.7	69
Pelvis cup tit	85.7	90.8	87.7	94.4	90.3	86.6	80.5	80–95	89.7	90
Pelvis cup	78.8	83.4	80.6	88.4	86	87	80.3	78–89	84.8	85
Rasp. femur	95.4	94	85.4	97.7	101.4	88.1	88.3	85–102	95.9	96
Femur stem	79.4	78.3	81.2	83.6	86	81.1	81.4	78–86	82.3	82
Ball fix	77	68.7	68.9	69.8	66.3	75.6	64	64–77	72.3	72

*Note.* Column names refer to the date of observation and order of surgery observed.

**Figure 7:**
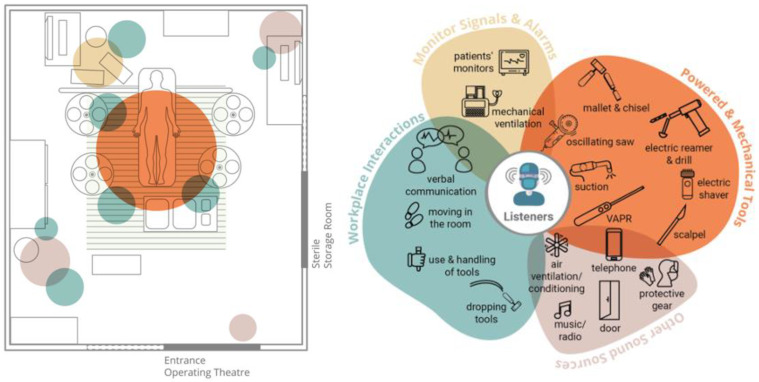
The sound map of an OR with the acoustic impact of sound events.

Figure 7 presents the most commonly found sound sources in our observations and their location within the operating room. Power tools (orange circle) are used around the patient and have a high impact on the operating table area. Conversations (turquoise circles) are spread around the room. Medical alarms (yellow circle) from monitoring devices are behind the patient close to the anaesthesiologist. There are a variety of other sound sources (pink circles) from interactions with the environments spread around the room. The most commonly used loud devices in our observations were the suction device, oscillating saw, mallet and chisel and medical alarms.

### 3.3. Task Analysis

**Figure 8:**
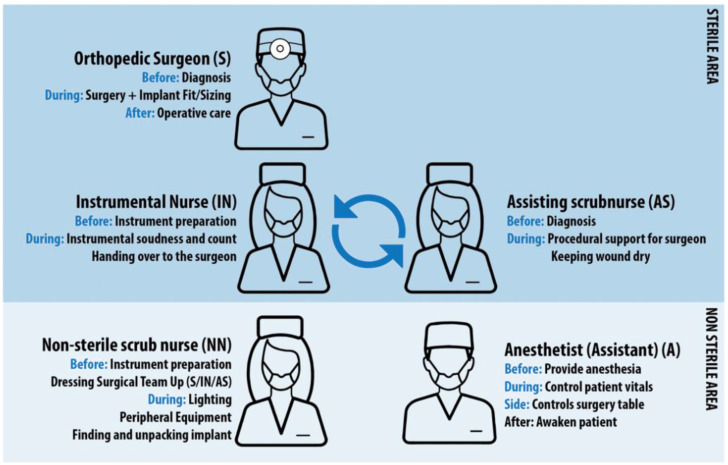
Task analysis of OR teams and their responsibilities.

During the total hip replacement surgery, a team of five people worked to replace the natural hip successfully. A team consisted of a surgeon, two operating assistants (sterile scrub nurses), one non-sterile nurse and an anaesthesiologist or anaesthetic assistant. All of them have a certain responsibility within the operation room (Figure 8).

**Orthopaedic Surgeon (S).** The surgeon is responsible for the diagnosis of the patient preoperative. Preoperative diagnosis leads to templating of the replacement hip, so the surgeon is preparing the operation beforehand. During surgery, the leader of the surgical team and responsible person for making decisions on the patients’ health, safety, sizing of the implant and fitting it into place. After surgery s/he is there to provide the patient with postoperative care and the necessary treatment such as rehab. All actions done by the surgical team, are given approval by the surgeon, if there is no approval, no action is taken.

**Sterile scrub nurses.** These two types of operating assistants support the surgeon during procedure from within the sterile area. Instrumental Scrub nurse (IN) faces the surgeon during operation, hands instruments to the surgeon and (dis)assembles them after actions taken by the surgeon. Next to that s/he prepares the surgery together with the assisting scrub nurse, by laying out and assembling all necessary equipment. The instrumental nurse also monitors sterility. S/he checks all instruments for soundness and usability. Furthermore, s/he is jointly responsible for the counting of the gauze and used instrumentation. Assisting scrub nurse (AN) stands next to the surgeon during the procedure and support the surgeon to give him the best view the operative field by keeping the wound dry with the aid of gauze or suction. Next to presenting the incision site in the best way possible s/he helps with disinfecting and covering the operative field. Occasionally the assisting nurse can suture the incision or wound after surgery.

**Non-sterile scrub nurse (NN).** This assistant prepares the operation together with the instrumentation assistant. They are not part of the sterile operating team. They do all the non-sterile work in relation to the surgery. They prepare all sterile materials before and during the operation and helps with dressing the sterile team. At the start of the procedure, they help with the positioning of the patient on the operating table. During surgery they ensure the connection and adjustment of all peripheral equipment and the correct illumination/lighting of the operating area. In addition, they are the ones finding the implant in stock and opening the packaging. After this they hand it over to the assisting scrub nurse.

Non-sterile nurses and sterile operating assistants rotate function between each surgery to ensure acerbity during procedure to assist surgeon as best as possible.

**Anaesthesiologist (A)/Anaesthetic assistant.** The anaesthesiologist is the doctor specialized in providing the patient with anaesthesia and pain relief medicine. During surgery responsible for monitoring vital functions such as blood pressure, heart rate and blood loss before, during and after surgery. In addition to the anaesthesiologist, there an anaesthetic assistant. Together they give the anaesthesia to the patient and control its values during the operation. The anaesthetic assistant will guide the patient, monitor its vital functions and will be present during the entire operation. Extra responsibility that the anaesthetic assistant has during surgery is the remote controls of the surgery table, by raising, lowering or tilting the table on surge- on request.

To conclude, the task analysis based on our observations in Figure 8 indicates that both orthopaedic surgeon and instrumental nurse are the people who handle the instruments and tools which cause loudest sounds level during surgery. Thus, surgeons and instrumental nurses are the main sound producers as they are the ones that use, check and prepare the instruments before, during and after the procedure.

### 3.4. Information Flow

The team is divided into sub-teams: The ‘sterile’ medical staff is responsible to perform the surgery (e.g., surgeon, operating assistant, and resident). They stay in the sterile or aseptic area (marked on the floor) during the entire surgery. The ‘non-sterile’ medical staff consists of those team members who are located outside the sterile area (e.g., circulating nurses and anaesthesiologists) [99]. They are responsible to take care of the patients’ well-being during the surgery (e.g., anaesthesiologists) and to support the surgical team (e.g., circulating nurse). Some surgeries also require other professions, such as radiologists [100] or other individuals present (e.g., researchers, medical students, etc.).

**Figure 9:**
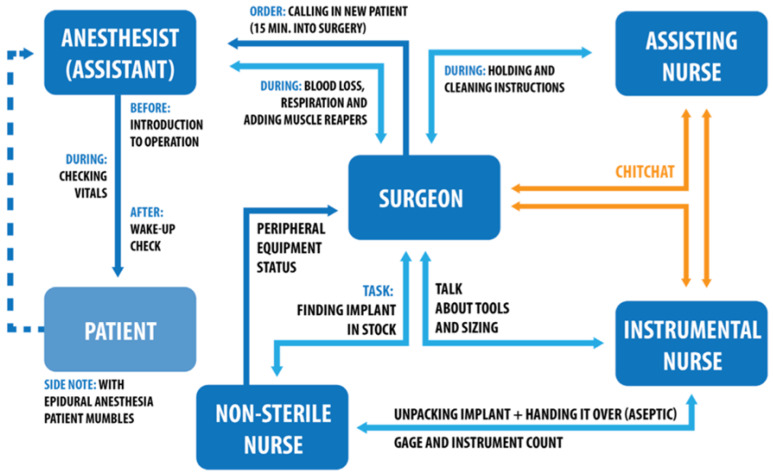
Information flow diagram within the OR team members.

Information flow can be seen as movement of communication between people and systems. In Figure 9, we present the flow of information from observations within the operation room. The auditory information transfer is either interrupted or stopped during the usage of (powered) instruments. Staff and surgeon do not verbally or auditorily communicate at these moments owing to the temporary interruptions to hearing. Thus, the sound of the surgical tool dominates the workflow. In these moments hand gestures were enough to spark an action in the operation room. For example, the surgeons open their hand and the surgical assistant places the next tool in their palm, that is the level of habituation they have working together. In that case, hand gestures or body language contributes to the flow of information. Moments of verbal communication during surgery and the exchange of significantly import information such as patient vitals and implant sizing only applies to a very small part of the surgery. A total of 8 min important information gets exchanged against 30 min of total surgery time and the remaining exchange of verbal information flows are unrelated to direct patient care and can be seen as chitchat. Circulating nurse (in non-sterile area) supports the whole team (needs to pay attention to anaesthesiologist, as well as operating team and people who might enter the operating room during surgery) and sterile nurse (or called scrub nurse) works directly at the operating table, interacting with different sounds than the circulating nurse (as s/he is for instance passing instruments to surgeon).

Surgery related verbal communication within the OR teams added up to approximately 8.5 min of surgery time (out of 30 min in total for THA procedures). The OR team talks about patient vitals, sizes of instruments and types of equipment, and instructions to the assistant or interns. Operation room staff have to communicate at speech levels of 90–100 dBs, measured average decibel was around 80 dB and during peaks at levels of more than 130 dB(C), which is painful and can permanently damage one’s own hearing.

In the observed procedures, surgeons, the surgical assistants and scrub nurses had many years of experience as part of their medical specialization. Moreover, they have been working together for an extensive period of time, so they are used to each other’s ways of communication, know the routine and are well-coordinated. The surgeries visited can almost be seen as assembly line work. For example, even before a surgeon is finished using an instrument and without asking, the following instrument is presented thanks to the established workflows. Yet, we also observed that nurses are actively listening to the sounds of surgery. There are two possibilities that explains how sound is part of the information flow. First, OR team members actively listen and predict tasks needed for the surgery using sound as information; secondly, when it is loud and noisy, they miss relevant auditory information, but compensate it with non-verbal communication skills but at a cost on their perceptual system.

### 3.5. Auditory Cues and Instrument Feedback

It is well-known that surgeons use sound cues produced by surgical tools to validate if the procedural steps are performed successfully. To find out how they interact with sound emitted by surgical instruments and tools, we first analysed how perceptually noticeable sounds would be in terms of their loudness. Our analyses did not consider the frequency content of the sound (while we know that frequency changes a result of a.o. timbre, pitch and distance) as we only measured the sound levels in more detail.

The variation in sound levels (i.e., intensity of energy in sound production) plays a critical role in noticeability [101]; noticeability by sound levels starting from 3 dB difference between two sounds, clearly noticeable for 5 dB difference, half or twice as loud for 10 dB difference, and much quieter or louder for 20 dB difference. Changes in sound level during surgery and surgical steps are just noticeable for hammer blow or clearly noticeable. Yet, the role of auditory cues remains controversial not only by the fairly small changes in sound levels but also the constant high-level of background music in the operating room which makes detecting small changes in sound levels nearly impossible. Music can bring the OR decibel level up to 87 dBs or more next to the already considerable ambient noise levels with suction pumps, alarms, mechanical ventilators and others. Next to that music can have a detrimental effect on surgical performance, especially among less experienced surgeons. In randomized controlled trials of novice surgeons, music during training procedures caused distraction and impaired performance [102]. When combining just perceptible increases of sound level with the music playing in the OR, sound cues are most likely not used by the orthopaedic surgeon. To demonstrate, in one of the observed surgeries the surgeon was striking the broaching handle on the beat of the music. Thus, music took over the attention of the surgeon and entrained them for using the mallet in a rhythmic way and the mallet sound was adapted to the other sound events in the OR demonstrating the adaptable nature of listening. However, we are unsure why surgeons keep in time with the music and whether it was to monitor the number and speed of the strikes of the mallet (i.e., to signal than a blow had been struck).

Our measurements and the literature (see Section 3.2) indicate that in orthopaedic surgeries not all sound cues can be readily used by the OR team as the sound level changes during surgical steps and only a few sounds are clearly noticeable by their loudness (see Table 1 and Figure 6) or sounds have to be at their peak level to be easily noticeable (e.g., rasping or hammer blow or alarms that are set to be louder). Making alarms more noticeable by loudness is also unacceptable for healthcare providers in critical care as feeds more alarm fatigue. Subtle sounds such as incision will be very difficult to be noticed by loudness in current circumstances with music added or when each blow of the hammer is above 125 dB probably causing temporary hearing impairment or loss [96,97,98]. However, the behaviour change of the surgeon to the beat of music is a promising finding and an indication that sounds can guide interaction within the OR.

### 3.6. Sound as a Tool for Surgeries

During and after our visual observational session we had semi-structured interviews with the OR team members which resulted in insights into how surgical sounds are part of the surgical tasks. We interviewed representatives of all OR team members (Orthopaedic surgeon/Instrumental nurses/Assisting scrub nurse/Non-sterile scrub nurses and Anaesthetic (assistant)). The following questions were asked: What is your experience with the sound emitting tools in surgery? Is sound used in surgeries? What are the most harmful or annoying sounds? We also asked follow up questions such as whether the team members use ear protection and why (not).

**Figure 10:**
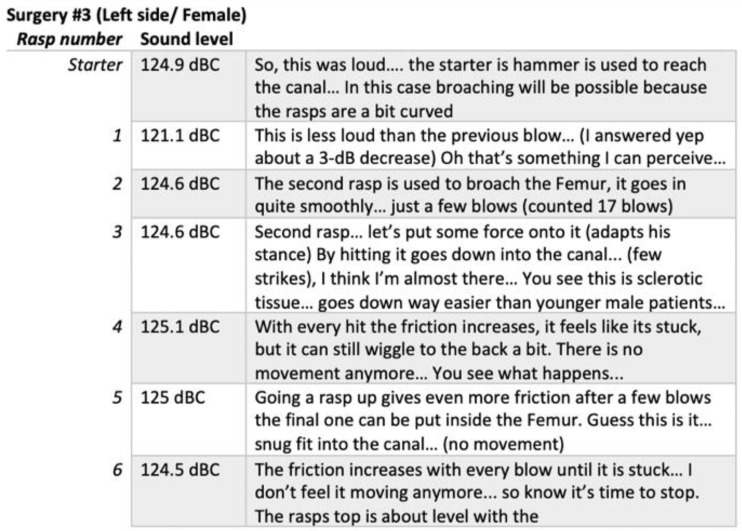
An excerpt of conversations (between the researcher and the surgeon) during the surgery demonstrating a surgeon’s awareness of the loudness of the hammer blow and the physical evidence with the dB(C) measurement that the second blow is measured to have a lower loudness level by 3.8 dB.

Especially surgeons and anaesthesiologists have explicitly mentioned during a talk-aloud as part of the observations that they use sounds as input for surgery and they follow the loudness of the hammer blow or the alarms of the patient monitors (See Figure 10). Hammer blows (i.e., the use of surgical mallet) are perceived as the most malicious sound with its loud peaks and oscillating saw as the most annoying as it is sharp and high-pitched. Some said they suffer from physical pain and headaches after a THA surgery. While other OR team members ‘complained’ about the mallet sound, surgeons came up with ideas and solutions how to improve the sound quality. Some of these solutions were mechanical design solutions, others were using a sound dampening material in between two metal materials that hit (mallet and cap), some use earplugs for protection. Surgeons’ finding a solution for sound design suggests that surgeons are aware of the use of sound and its potential and harm. Surgeons also showed awareness to how sound changes when they are actively engaged with power tools as they described the changing pitches, or changing loudness in the sound quality. This also shows that surgeons actively use sounds.

**Figure 11:**
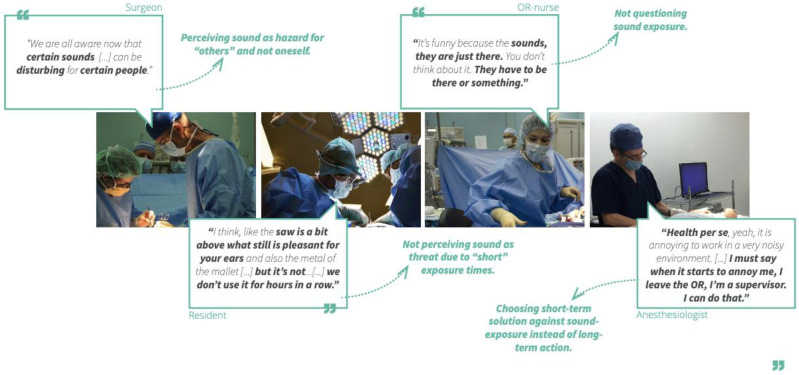
Quotes of medical staff, including the researchers’ interpretation. Photos (retrieved freely from Pixabay) in this artistic representation do not belong to our study.

Many opposed using earplugs as they potentially block verbal cues such as commands and auditory cues that are needed for the surgical tasks. It was mentioned that surgeons’ actions may be louder when they use earplugs. Many pause conversations during loud sounds. Anaesthesiologist and anaesthetic assistants indicate that patient monitor alarms may signify a calamity and cannot be missed. Surgical sounds are also considered as communication and many do not wish it to be silenced. Next to sounds surgeons especially use tactile feedback in addition to sound to feel whether the metal cap is well inserted by the mallet’s hammer blow. In Figure 11, we exemplify some key comments of the OR teams within a visual diagram.

### 3.7. Types of Listeners within the OR

In orthopaedic operating rooms, several different professions work side by side. As tasks differ, listeners are prioritizing individually to which degree they pay attention to the auditory information that arrives from different sound sources. To be able to understand the listening behaviour of the OR team members, we used Truax’s categorisation of listening types [50] and identified five listening attentions (instead of the original three categories by Truax) in the OR. Truax initially proposed three types of listening attention and our study context offered two more that are adjacent to the original three from the perspectives of the patient as the ‘exposed-listening’ and of the surgeon as the ‘listening-in-action. Thus, we propose the following five different types of listeners: “exposed-listening” by **inactive listeners**, “background listening” by **passive listeners**, “listening-in-readiness” by **active listeners**, “listening-in-search” by **sound users** and “listening-in-action” by **sound producers**.

Considering an acoustic biotope as a shared functional acoustic space, and using the information gathered in the previous sub-sections, the different roles of these listeners can be explained. Patients are **inactive listeners** despite being in the center of all attention during a surgery. If they are fully sedated, they may not be conscious about their sound environment. If patients are partially sedated (e.g., from waist down), they can revert their attention to the sound events taking place but will have no control for change making patients exposed to sound and therefore inactive. **Passive listeners** (typically circulating nurses) often have another task to focus and attend, therefore they listen to the background for scanning any relevant information but do not react to majority of the sound events. **Active listeners** (typically scrub nurses) listen in readiness to be able to support the main tasks of the surgery following the surgical procedures by sound and making decisions when to support mainly the surgeon. **Sound users** (e.g., surgeons, anaesthesiologists) typically rely on sound-induced action in an acoustic biotope as they actively search for auditory cues for the benefit of their task at hand and use these cues to complete tasks. In the case of OR, surgeons use the sound of mechanical tools (e.g., mallet) or power devices (e.g., saw, drill) as continuous feedback to navigate through the different steps of surgical procedures. Then, sound becomes the surgical tool to work with. **Sound producers** (typically surgeons, instrumental nurses and anaesthesiologists) not only listen but also emit sound as part of their assigned task when they talk to eachother, use mechanical and electric surgical tools, enable alarms, etc. Their attention to sound events and quality could be at a heightened level enabling their sensory-motor skills facilitating sound-induced action and reaction. In facts, sound producers become a part of the operating table contrasting their position with the patients who are exposed to sound events triggered by surgeons; therefore, they actively contribute to the sound environment as well as actively listen to it.

**Figure 12:**
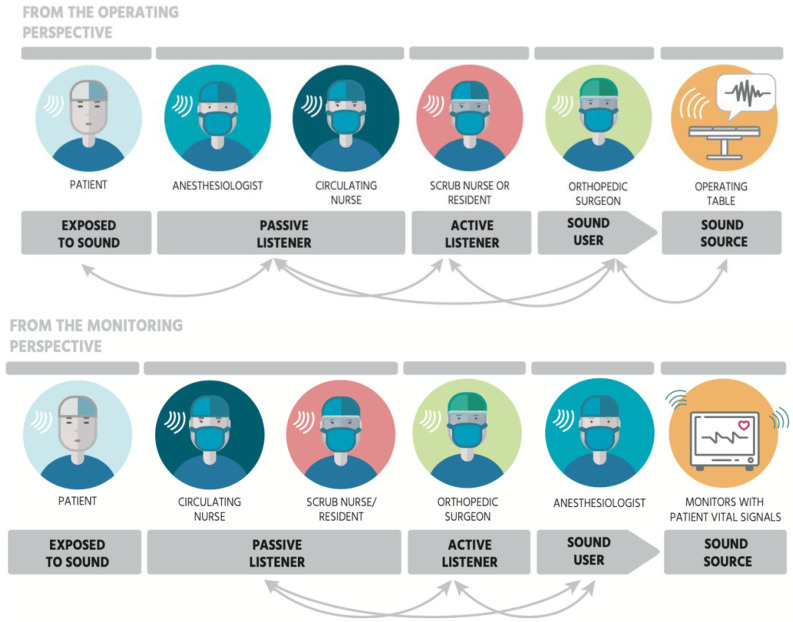
Listening attention fluctuates within the OR and is shown by arrows in the figures. Listeners switch attention and change roles in workflows according to the task at hand, e.g., from the operating perspective (i.e., surgeon) in the top image and from the monitoring perspective (i.e., anaesthesiologist) in the bottom image.

All these listeners engage in sound-induced action to a varying degree in an acoustic biotope. Two streams of listener hierarchies were observed (see Figure 12), depending on the main focus of listening: the perspective of performing the surgery (e.g., surgeon) or the perspective of monitoring the patient (e.g., anaesthesiologist). For instance, both the surgeon and the anaesthesiologist are sound users focusing on different tasks. The anaesthesiologist primarily listens to the patient’s vital signs, while the surgeon primarily listens to the sound events at the operating table (e.g., feedback from tools). However, OR team members can choose to change their attention based on the event, situation, task at hand, and their needs, which will be explained below.

Although a general distinction between the listeners can be made, listening attention may fluctuate according to the situation within the surgery (indicated in Figure 12 as arrows). For example, scrub nurses performing a specific task in the procedure will become sound users themselves, but will most likely switch back into the active listening after the task has been completed. The only listener not fluctuating their attention are the patients, because they are (mostly) sedated. When sedated, their ears are still “exposed” to the sounds and their hearing system actively captures sounds. Patients’ inactive listening deserves a separate investigation as their listening may be shaped by other factors (e.g., anxiety, fear, lack of familiarity) despite having the least control over the sound events.

## 4. The Main Elements of Acoustic Biotopes of ORs

Our study demonstrated the complex nature of OR sounds and their role in the surgical tasks. We bring all the insights together to compose the basic components of an acoustic biotope fitting for OR listening experiences. Our observational study as well as the extensive literature review have brought forward many notions which we need to consider to understand how OR team members interact with OR sounds or conduct surgery with the help of OR sounds. Below we will summarise our findings with the introduction of the terminology (i.e., acoustic space, acoustic environment, bio-acoustic space and acoustic biotope) proposed by Aartsen, Johannessma, and Smolders [12,13]. Furthermore, the insights gained in this section will be relevant to OR teams, patients, clinical managers, designers for healthcare, clinical technologists and policymakers who are active users of OR sound, affected by cacophony, or influential for structural change in healthcare.

### 4.1. Listeners as Actors in an Acoustic Space

Acoustic spaces offer a range of sounds but acoustic biotopes only exist if there are listeners with an intention to interact with their environment. In this paper, we distinguished these actors/listeners as part of their professional roles and observed their tendency to capture some or all sounds. Thus, to a certain extent, these professional roles also define how actors listen in an environment as part of the surgical workflows. However, how these listeners (i.e., actors) relate to sound may also derive from their own belief systems, prior experiences, years of experience as a professional, cultural background and sensory sensitivity. These socio-cultural differences within and between listeners effect the listening experience and should be studied separately to further understand the prior conditions that shape listening in the broader context of the acoustic space.

### 4.2. Sound Producing Events in the Acoustic Environment

The are many different categories of OR sounds (e.g., alarms, speech, power tools, instruments, music) and OR teams are expected to deal with them efficiently for the success of the surgery. However, these sounds compete with each other in terms of noticeability. Currently, only the loudest sounds seem to capture the listeners’ attention. In order to provide a more functional workplace, it would be beneficial to organise sound events in an acoustic biotope in order to allow for individual sounds to be present and noticeable. While music may have an encouraging effect for the surgeon, it also masks audible alarms, subtle cues in other interaction sounds and verbal communication which are already hindered by surgical masks. The same observation is valid for the sound of suction devices and oscillating saws, all of which produce loud and broad frequency sounds that are likely to mask other subtle cues which may also come from people and their expressions. Here, is a plea to device manufacturers to develop products that emit biotope-friendly sounds.

### 4.3. Locals of Sound and Localizability in the Acoustic Environment

As ORs are rather small acoustic environments, saliency of sound events brings its own challenges. In the operation room most of the noisy activities are found inside the sterile area and come from the users of surgical tools and human-instrument interactions. Power tools, and music (and to some extent verbal communication) emit the loudest perceived sounds within the OR. The surgical table is the loudest place yet sounds can come from different angles and the OR team members will have a tacit knowledge regarding the existing of objects, visible or invisible to them, with the help of locating the sound source. Moreover, within the OR environment, sounds that are relevant to the surgery and sound that are not relevant to the surgery can be easily filtered out. It needs to be mentioned that non-surgical sounds (i.e., any sound in the acoustic space of the hospital) can be equally relevant for the success of the surgery (e.g., fire alarm of the hospital).

### 4.4. Listening Capacity within the Bio-Acoustic Space

Not all OR listeners possess the same listening capacity as a result of individual differences in hearing (e.g., hearing loss happens often amongst orthopaedic surgeons), physical position in the room, role in the surgical tasks and consequently listening attention. Moreover, each listener seems to train their hearing according to their designated tasks and tune in to the sounds that are relevant to them. Such training is a result of repetition surgical procedures or years of experience. The experienced sound therefore is dependent on the bio-acoustic space of the listeners.

### 4.5. Common Goal within the Acoustic Biotope

Acoustic biotopes of ORs should be considered as functional professional settings in which OR teams strive for the success of the surgery and patient safety. Thus, this notion frames their way of interaction within the OR environment. OR teams are professionally trained people and need years of experience to be able to work independently in a team. All the surgical procedures are well rehearsed and therefore familiar. Sound takes part in these routines and procedures. Thus, OR sounds are engraved in the order of surgical task and their probability of occurrence is dependent on the timing of the tasks and the actor. The OR surgeon, nurses, and the anaesthesiologists all have a common understanding and work in symbiosis. The sound’s role in this should be strengthened for more effective, perceptually comfortable, and healthy workspaces.

### 4.6. Interactions with and via Sounds in the Acoustic Biotope

In the acoustic biotopes, many interactions take place either directly with OR power tools, with other team members or the environment via sound. Thus, acoustic biotopes should facilitate all these interactions and should be place in which species (i.e., OR team members) thrive and find opportunities for sound-induced action. Team members should be able to acknowledge sound as a complementary tool aiding surgical flows and trained to use sound to their benefit. Yet, we have not conducted a behaviour analysis on facial expressions and non-verbal communication that evidences the absolute use of sounds as cue. Such a study will bring in more introspective insights into the implicit use of sound for surgical tasks.

## 5. Key Takeaways for Acoustic Biotopes

Our study provided a holistic, multi-layered and yet a listener-centric view on the organisation of complex spaces. Our results are ecologically relevant and supported by sound measurements, sound recordings, interviews, observations and existing literature. Accordingly, we establish that acoustic biotope is an active and shared sound environment with entangled interactions and sound-induced actions taking place in a specific space that has a critical function. Below, we summarise our first take-aways:

- Acoustic biotopes of socio-technological environments should be considered as functional professional settings in which teams collectively and individually strive for the success of the mission and the local tasks through sound-induced action.
- Listening in highly functional environments is an individual experience and is influenced by hearing function, physical position and role in an environment, and the task at hand. Other sound events in the environment and their spectro-temporal structure will influence hearing as well but would be considered as an external factor that could affect all listeners at a varying degree.
- Acoustic biotopes facilitate all kinds of interactions with sounds, system tools and team members via sound, and must offer opportunities for sound-induced action for all types of listeners.
- There are many different sound sources in the socio-technological environments and all of which can be used by different actors in different ways. Different uses of sound should be defined in relation to listener actions.
- In acoustic biotopes, there are different locals of sound events with different sound intensities and complexities as a result of the concentration of tasks. These sound zones are rich with sound-induced action and should be well organized.
- Power tools, music, and verbal communication emit the loudest perceived sounds within the acoustic biotopes and some of these sound sources can be harmful for listeners.
- There is a range of active, passive and inactive sound listeners as a function of their attentive state and listeners as sound user as well as sound producers within the acoustic biotope. It is important to define these dynamics of how and when listeners switch attention and change the way they listen for assigning relevant tasks.

## 6. Conclusions

The design research community currently lacks knowledge to establish theories/models explaining the extent to which listeners differ in their experience and interpretation of the sound events in the shared sound environment and how such differences influence their individual tasks at hand and success of the shared goal. Such knowledge is required for creating functional workspaces. So far, the majority of the methods into sound perception and action tackled specific relationships between one type of listener and one type of sound often isolated from the environment. When complexity is at hand, researchers investigated ecologically relevant sounds as a whole or looked for collective responses to them as in soundscape perception studies. As such, to be able to establish the notion acoustic biotopes that recognizes the plurality of sound producing events and listeners, we applied an ecologically relevant novel approach that is listener-centric and embraces the complexity of multiple pathways between sound sources and listeners looking for patterns, regularities, laws, and structure in the sound-action coupling in context.

**Figure 13:**
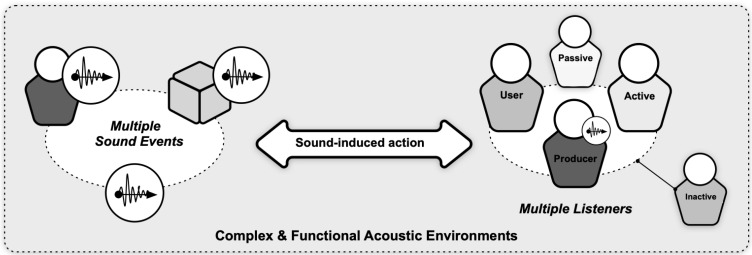
Challenges of complex acoustic environments with multiple sound sources and listeners.

Our study into the ‘acoustic biotope of an OR’ provides us with a complex and multi-layered phenomenon to further analyse as the cause-effect relationship between sound and listener is not direct in a shared acoustic space and there are external (latent) factors that determine the outcome of the perception-action trajectory. Thus, we observe two empirical challenges to be further studied for the acoustic biotopes of socio-technological environments (Figure 13): First, there are multiple sound sources (e.g., medical devices, surgical tools, speech, music) that require the attention of teams which hear sounds in a sequence and have to detect the useful ones. Secondly, there are categorically different listeners in the OR that are exposed to or interact within the acoustic space (Figure 13). Some listeners (e.g., surgeons/nurses), are active contributors to the sound environment and use sound continuously for important tasks based on different listening modes. Other listeners (e.g., anaesthesiologists) are at the periphery of the immediate surgical tasks and focus on self-selected sounds (e.g., audible alarms) to support the assigned tasks. Yet, although some of these relations are obvious from the roles and equipment that the actors use, others may be worth discovering. For example, can we facilitate acoustic conditions for the OR team members so that they are able to direct their own actions in harmony with the surgeon’s efforts? Can we support sound-aware workflows through design of appropriate machinery/equipment or space? Can we train the professionals to be aware of dynamics of sound use? Sound interpretation, then, varies and is highly dependent on the type of listeners and their actual needs. In future research, we will use a series of complementary methods focusing on descriptive analysis of the sound environment, empirical explorations of cause-effect between sound and listeners’ responses, and comparative study in simulators to measure the effect of new sound environments on professionals. However, most importantly our study showed an urgent need for studying the conditions for ‘sound-induced action’ within, and ‘actionability’ as a measure for the quality of, shared and functional acoustic spaces.

## Figures and Tables

**Table 2:** Registered dB(C)—LCpk during surgeries.

dB(C)-LCpk	21.10/2	21.10/5	01.11/3	01.11/2	14.11/1	14.11/2	Range	Average (log)	Set Average
Incision	82.6	85.5	87.2	92.1	89	84	82–92	87.9	88
Saw (osc)	100.2	101.4	106.1	105.6	95.7	95	95–106	102.5	102.5
Clean-up/suction	93.4	88.2	90.6	86.8	94.9	95.1	86–95	92.6	92
Reamer (elec)	91.7	94.7	88.5	95.2	90.2	91.9	88–95	92.7	93
Pelvis cup tit	123.4	124	123.6	124.7	124.2	124	123–125	124	124
Pelvis cup	119.3	123.6	117.4	119	121.7	114.8	114–124	120.2	120
Rasp. femur	121.5	119	125.1	124.9	122.1	121.3	119–125	122.8	123
Femur stem	116.2	114	122.7	120.6	116.6	118.4	114–121	119.1	119
Ball fix	97.2	95.2	92.1	98.5	114.5	108.4	92–115	107.9	108

*Note.* Column names refer to the date of observation and order of surgery observed.

## Data Availability

Sound recording data and interview data are anonymously available upon request.
